# Validation of the World Health Organization Disability Assessment Schedule‐II for Measuring Women With a History of Potentially Life‐Threatening Maternal Conditions at Six Months Postpartum in Tigray, Northern Ethiopia

**DOI:** 10.1002/hcs2.70054

**Published:** 2026-02-11

**Authors:** Fitiwi Tinsae Baykemagn, Girmatsion Fisseha Abreha, Yibrah Berhe Zelelow, Alemayehu Bayray Kahsay

**Affiliations:** ^1^ College of Medicine and Health Sciences Adigrat University Adigrat Ethiopia; ^2^ College of Health Sciences Mekelle University Mekelle Ethiopia; ^3^ Department of Obstetrics and Gynecology Mekelle University Mekelle Ethiopia

**Keywords:** functioning and disability, reliability, severe maternal morbidity, validity, WHODAS‐II

## Abstract

**Background:**

The World Health Organization Disability Assessment Schedule 2.0 (WHODAS 2.0) is a popular tool for evaluating functioning and disability in a range of population demographics and medical situations. However, very little is known about the WHODAS 2.0's validity and reliability, particularly when dealing with potentially life‐threatening maternal conditions (PLTCs). The aim of this study was to evaluate the validity of the WHODAS 2.0 Tigrigna version.

**Methods:**

This cross‐sectional study was conducted in Tigray, northern Ethiopia, from December 15 to 20, 2023. Following translation and back translation, women who had experienced PLTCs during a recent pregnancy, childbirth, or postpartum period were administered the 36‐item WHODAS 2.0 in Tigrigna version 6 months after the childbirth. In total, 121 women with a history of PLTCs participated. Cronbach′s α was used to evaluate internal consistency in all six WHODAS 2.0 domains, while Spearman′s correlation coefficient was used to evaluate convergent validity. With confirmatory factor analysis, construct validity was also examined.

**Results:**

All domain scores of the Tigrigna version of the WHODAS 2.0 indicated excellent internal consistency (*α* = 0.917–0.978 for 36 items and *α* = 0.874–0.940 for 12 items), while the Cronbach′s α coefficients for the summary score were 0.981 and 0.952 for 36 and 12 items, respectively. The convergent validity between the 36‐item and 12‐item WHODAS 2.0 showed a strong correlation between similar constructs (*r* = 0.909–0.981).

**Conclusion:**

Despite the small sample limitation, the WHODAS 2.0 tool adapted to the Tigrigna version indicated an acceptable reliability and validity and therefore could be applied to women with a history of PLTCs at 6 months postpartum.

## Graphical abstract

The World Health Organization Disability Assessment Schedule 2.0 (WHODAS 2.0) of the Tigrigna version has strong internal consistency (Cronbach's *α* coefficients = 0.98). The convergent validity between the 36‐item and 12‐item WHODAS 2.0 indicated a strong correlation (*r* = 0.909–0.981) within similar domains. The WHODAS 2.0 Tigrigna version is reliable and valid to measure the disability level within severe maternal morbidity patients.

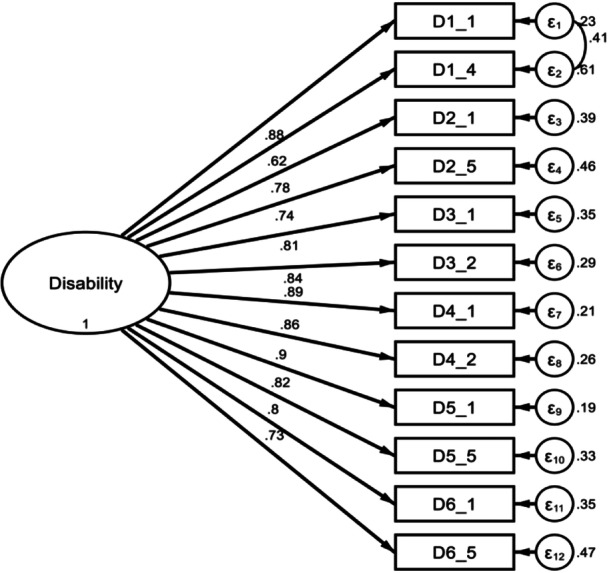

**Abbreviations**
CFA: confirmatory factor analysis
CFI: comparative fit index
CVI: content validity index
D: domain
ICVI: item content validity index
KMO: Kaiser–Meyer–Olkin test
PLTC: potentially life‐threatening conditions
*r*
: Spearman′s correlation coefficient
RMSEA: root mean square error of approximation
SCVI: scale validity index
SD: standard deviation
SRMR: standardized root mean residual
TLI: Tucker Lewis index
WHO: World Health Organization
WHODAS‐2: World Health Organization Disability Assessment Schedule‐II

## 1 Introduction

Potentially life‐threatening maternal conditions (PLTCs) are the most common cause of maternal mortality worldwide, particularly in low‐and middle‐income countries [1, 2]. According to a systematic review, Ethiopia has the highest prevalence of PLTCs (17.55%) [3]. PLTC is severe maternal morbidity that includes obstetric hemorrhage, severe management indicators, hypertensive disorders, and other systemic disorders [4, 5]. In addition to mortality, PLTCs have a substantial impact on maternal morbidity, which leads to mental and physical impairments that impact everyday functioning and quality of life, some of which may be permanent or irreversible [6, 7]. Among women aged 15–44, these conditions also rank as the third leading cause of disability‐adjusted life years [8].

The World Health Organization's (WHO) report stated that 15% of the world′s population has a disability [9], but in Ethiopia the prevalence was 0.74% in 2022 [10]. This discrepancy may arise from Ethiopia′s narrower definition of disability, which focuses on difficulties in daily activities and excludes restrictions in social participation [10, 11], compared to the WHO's comprehensive definition that includes impairments, activity limitations, and participation restrictions [12]. Information about disability and functioning is becoming more important in the field of public health. Its significance as a third health indicator, alongside morbidity and mortality, is being discussed. These three health indicators supplement one another to give the health system more precise information [13]. Addressing the full spectrum of maternal morbidities and related disabilities is one of the five strategic objectives of the Sustainable Development Goal 3, which aims to end preventable maternal mortality [14].

The 2015–2020 Health Sector Transformation Plan and other national policies and guidelines in Ethiopia place a strong emphasis on enhancing maternal health by increasing access to emergency obstetric care and skilled birth attendance [15]. These guidelines, however, lack specific protocols for managing long‐term functional disabilities resulting from PLTCs and instead focus primarily on preventing mortality. In settings with a lack of resources, such as Ethiopia, where access to rehabilitation is limited, the functions of healthcare professionals, community health workers, and rehabilitation services in addressing these disabilities are still not well understood [16]. This disparity is particularly significant in low‐and middle‐income countries, where health systems deal with issues like poor infrastructure, a shortage of skilled workers, and socioeconomic barriers, all of which increase the burden of maternal morbidity and disability [17, 18].

Validating the World Health Organization Disability Assessment Schedule‐II (WHODAS 2.0) for PLTCs is an important step toward understanding the functional impact of severe maternal health conditions, particularly in postpartum women. The results of this study can influence clinical practice, guide policy development, and support initiatives aimed at improving the health and well‐being of women who suffer from severe maternal health problems. To address this, we conducted a methodological investigation that looked at the psychometric properties of the WHODAS 2.0 Tigrigna version instrument for those with PLTCs to make sure the translation and adaptation were successful. This study aimed to evaluate the validity and reliability of the WHODAS 2.0 tool in women who experienced PLTCs at 6 months postpartum.

## 2 Methods

### 2.1 Study Participants, Sampling, and Data Collection Procedure

A cross‐sectional validation study was conducted using the hospital′s registration book and medical chart to identify all women who had a history of PLTCs from June 10 up to July 10, 2023. Participants were women assessed 6 months post‐PLTCs in Tigray, northern Ethiopia. After extracting the list of study participants from the registration book, women were contacted via telephone and visited at their homes for data collection between December 15 and 20, 2023. The WHODAS 2.0 tool was administered via interviewers to 121 women who had a history of PLTCs.

### 2.2 Measurements

The WHODAS 2.0 is a multifaceted, comprehensive disability assessment tool. Six domains comprise the full WHODAS 2.0 (36 items): the first domain is called “Cognition” (six items); the second is called “Mobility” (five items); the third is called “Self‐care” (four items); the fourth is called “Getting along” (five items); the fifth is called “Life activities” (eight items: four items related to activities at home and four related to activities at work or school); and the sixth is called “Participation” (eight items). When time is limited, the 12‐item short‐form version can be used to quickly evaluate general functioning and disability in clinical and research contexts. S1 through S12 were the labels assigned to the 12 WHODAS 2.0 items. Two questions from each of the six domains in the 36‐item version comprise the 12‐item WHODAS 2.0's structure: “cognition” (D1.1 = S6 and D1.4 = S3), “mobility” (D2.1 = S1 and D2.5 = S7), “self‐care” (D3.1 = S8 and D3.2 = S9), “getting along” (D4.1 = S10 and D4.2 = S11), “life activities” (D5.1 = S2 and D5.5 = S12), and “participation” (D6.1 = S4 and D6.5 = S5). The degree of difficulty encountered during the previous 30 days is represented by the five possibilities for each item response: none = “0”, mild = “1”, moderate = “2”, severe = “3”, extreme/cannot do = “4”. If the person is neither working nor enrolled in school, the 36 and 12 items are lowered to 32 and 11, respectively. A higher WHODAS 2.0 score indicates greater difficulties in daily activities. The overall score ranges from 0 to 100 (“0” = no disability, “100” = full disability) [12].

### 2.3 Translation and Adaptation

The form was first translated into the Tigrigna language by the corresponding author, a maternity and reproductive health specialist, while taking into account the local community′s conceptual and cultural background. Experts in midwifery, nursing, gynecology, and public health evaluated the initial draft. The second draft was then back‐translated into English by a person who speaks Tigrigna and English proficiently. A third draft was produced following a comparison of the original and back‐translated versions.

### 2.4 Data Quality Control

We performed the following activities to ensure the quality of the data: (1) we provided 2 days of training to the supervisors and data collectors; (2) we closely monitored the data collection process; and (3) we used an electronic mobile application (Kobo toolbox) for data collection.

### 2.5 Data Analysis

For data analysis, we used Stata version 17 (StataCorp LLC, College Station, United States) and SPSS version 24 (IBM, Armonk, United States). To describe the socio‐demographic characteristics of the study participants, we used descriptive statistics. The data showed a considerable deviation from the normal distribution, as indicated by the Shapiro–Wilk test *p*‐value of less than 0.05. Therefore, in order to assess the relationship between the 36‐item and 12‐item WHODAS 2.0, we employed Spearman's correlation coefficient. Furthermore, we employed three methods to examine the data: content validity, construct validity, and internal consistency.

#### 2.5.1 Reliability

Internal consistency was evaluated using the Cronbach's alpha coefficient. Cronbach′s alpha coefficient was categorized as: excellent (*α* ≥ 0.9), good (0.8 ≤ *α* < 0.9), acceptable (0.7 ≤ *α* < 0.8), questionable (0.6 ≤ *α* < 0.7), poor (0.5 ≤ *α* < 0.6), and unacceptable (*α* < 0.5) [19]. To determine if individual items correlated to the same construct, the item‐deleted Cronbach′s alpha coefficients were also calculated. Item‐total correlations (correlations between each scale item and the overall score) were used to further analyze internal consistency. Spearman‐Brown scores, which are more effective at assessing the internal consistency of scales with only a couple of items, were also included in the internal consistency [20].

#### 2.5.2 Face and Content Validity

Initially, we used various kinds of expert opinions with experience in maternal health research (gynecologists = “1”, midwives = “3”, public health = “4”, and nursing = “1”) to evaluate the face and content validity. We also attempted to make sure that the experts had a clear understanding of and expectations for tool development. During a panel discussion, the experts offered valuable input for tool enhancement. Therefore, to encourage straightforward communication, we made a face‐to‐face approach through an expert panel discussion. Initially, the experts reviewed the item part (face validity) and concluded that the tool was suitable for assessing functioning and disability in the study region (Tigray Region). In the second step, the WHODAS‐2 tool′s content validity was checked to determine if it adequately evaluated the disability issue in the local (Tigray region) context. The item content validity index (ICVI) and scale content validity index (SCVI) are two components of the content validity index (CVI), which we used to evaluate content validity. Using a four‐level item rating system (1 = not relevant, 2 = somewhat relevant, 3 = fairly relevant, and 4 = highly significant), the CVI was determined based on the evaluation of nine experts in the disciplines of public health, midwifery, nursing, and obstetrics. While a score of three or four represented agreement, a score of one or two showed disagreement. According to the study, a CVI of ≥ 0.78 is acceptable when there are nine or more experts [21].

#### 2.5.3 Floor and Ceiling Effects

The floor and ceiling effects were evaluated by calculating the percentage of subjects who scored the lowest or highest results on the WHODAS 2.0 items. The floor or ceiling effects were detected if more than 15% of the participants gave the lowest or highest score, respectively [22].

#### 2.5.4 Construct Validity

Construct validity refers to how successfully an instrument measures the theoretical concept that it is intended to measure. We employed Spearman correlations to examine convergent validity between the findings of the 36‐item and 12‐item WHODAS 2.0 when there was skewed data distribution. The correlation levels were categorized as very weak (0–0.19), weak (0.20–0.39), moderate (0.40–0.59), strong (0.60–0.79), and very strong (0.80–1.00) [23]. We hypothesized a correlation between the 12‐item WHODAS 2.0 and the 36‐item WHODAS 2.0 disability assessment. The degree of disability on the 36‐item WHODAS 2.0 increases with the degree of disability on the 12‐item WHODAS 2.0 [12].

#### 2.5.5 Confirmatory Factor Analysis (CFA)

Exploratory or CFA can be used to assess construct validity [24]. Exploratory factor analysis is employed when a researcher wants to discover the pattern of answers; in these circumstances, the structure of factors is data driven, whereas CFA starts with a hypothesis about how many factors there are or which items load on which domain or factors there are [25]. We performed the CFA since functional disability has a known dimension. The sample size for this study was 121 participants. While the Kaiser–Meyer–Olkin (KMO) index (> 0.5) and Bartlett′s test of sphericity (*p* < 0.05) were used to assess sample adequacy and factorability, we acknowledge that the relatively small sample size may limit the statistical power and stability of the factor structure, potentially affecting the precision of model fit indices like root mean square error of approximation (RMSEA), standardized root mean square residual (SRMR), Tucker Lewis Index (TLI), and Comparative Fit Index (CFI). The acceptable model fit was determined at RMSEA < 0.08, SRMR < 0.08, TLI > 0.90, and the CFI > 0.90) [26, 27].

## 3 Results

### 3.1 Characteristics of the Study Participants

In total, the study included 121 participants who had experienced potentially life‐threatening maternal conditions during recent childbirth. The average age was 28.95 (±6.62) years. Most of the study respondents were 115 (95.04%) married, 88 (72.73%) housewives, and 80 (66.12%) lived in urban areas (Table 1).

**Table 1:** Socio‐demographic characteristics of the study participants in Tigray, Northern Ethiopia, 2024.

Variables	Categories	Data
Age (years)	Mean age	28.95 ± 6.62
Educational status	Did not attend school	19 (15.70)
	Primary (Grades 1–8)	19 (15.70)
	Secondary (Grades 9–12)	55 (45.50)
	Diploma and above	28 (23.10)
Place of residence	Rural	41 (33.90)
	Urban	80 (66.10)
A woman's main occupation	House wife	88 (72.73)
	Governmental employed	20 (16.53)
	Merchant	13 (10.74)
Marital status	Single	6 (5.00)
	Married	115 (95.00)

*Note:* Data are presented as mean ± standard deviation and frequency (percentage).

### 3.2 Face and Content Validity

To determine face and content validity, we consulted different types of experts. Experts followed structured steps to ensure clarity and orientation on validation expectations. They assessed the tool′s face validity, reviewing individual items for relevance and clarity. After evaluation, they concluded the instrument was suitable for assessing disability and functioning within the Tigray region's specific context. In the panel discussion process, the experts translated two WHODAS 2.0 items synonymously: “Getting out of your home” and “maintaining friendships” to “Move out of the yard/corridor” and “continuing friendships,” respectively. The second step, content validity, was ensured by providing an answer to the questions of whether the WHODAS‐2 tool was appropriate for evaluating the disability issue in the Tigray setting. The results of the content validity index from the nine participating experts were I‐CVI = 0.78–1.0 and S‐CVI = 0.96.

### 3.3 WHODAS 2.0 Domain Scores

The overall means (SDs) of the 32‐item and 36‐item WHODAS 2.0 were 34.60 (26.28) and 32.16 (24.96), respectively. The minimum disability means score was 12.65 in the ‘work/school activities' domain, and the maximum score was 41.78 in the ‘life activity/home' domain. The mean score for the 12‐item WHODAS 2.0 varied between 25.00 and 77.88 (Table 2).

**Table 2:** Descriptive analysis of the 36‐item and 12‐item WHODAS 2.0 scores.

	36‐item WHODAS 2.0 score					12‐item WHODAS 2.0 score				
Domain	Mean	SD	Median	IQR	Min–Max	Mean	SD	Median	IQR	Min–Max
Domain 1	31.06	25.81	25.00	45.83	0–87.50	25.00	11.18	31.25	18.75	0–43.75
Domain 2	39.87	25.70	40.00	37.50	0–95.00	50.00	22.36	62.50	37.50	0–87.50
Domain 3	27.89	27.42	25.00	50.00	0–100	58.17	34.63	75.00	62.50	0–100
Domain 4	30.57	29.74	25.00	50.00	0–100	58.00	33.34	75.00	62.50	0–87.50
Domain 5.1	41.78	31.80	25.00	43.75	0–100	77.88	27.68	100.00	50.00	25–100
Domain 5.2	12.65	25.93	0.00	0.00	0–93.75	62.50	21.50	75.00	25.00	25–100
Domain 6	36.23	28.58	25.00	50.00	0–93.75	62.50	20.91	75.00	25.00	25–100
Overall‐32 item	34.60	26.28	25.00	52.73	0–86.71	36.36	27.65	27.27	51.14	0–93.18
Overall‐36 item	32.16	24.96	22.22	47.91	0–84.72	66.17	25.08	78.40	47.73	15.95–93.18

Abbreviations: IQR, interquartile range; SD, standard deviation; WHODAS‐2, World Health Organization Disability Assessment Schedule‐II.

### 3.4 Reliability

Each of the following domains' Cronbach's *α* values showed excellent internal consistency in the WHODAS‐2.0 Tigrigna version: cognition (D1 = 0.965), mobility (D2 = 0.961), self‐care (D3 = 0.925), getting along (D4 = 0.965), housework (D5.1–4 = 0.978), work or school activities (D5.5–8 = 0.917), and participation (D6 = 0.976). The 36‐item WHODAS 2.0 had an overall internal consistency score of 0.981 (Table 3).

**Table 3:** Descriptive statistics and reliability of the WHODAS 2.0 Tigrigna version.

Domain (D)	Cronbach's *α*	Item‐total correlation	Cronbach's *α* if item deleted
Cognition (D1)	0.965	—	—
Item D1.1	—	0.904	0.956
Item D1.2	—	0.933	0.953
Item D1.3	—	0.907	0.956
Item D1.4	—	0.893	0.957
Item D1.5	—	0.871	0.960
Item D1.6	—	0.816	0.964
Mobility (D2)	0.961	—	—
Item D2.1	—	0.923	0.946
Item D2.2	—	0.870	0.955
Item D2.3	—	0.886	0.952
Item D2.4	—	0.897	0.951
Item D2.5	—	0.882	0.954
Self‐care (D3)	0.925	—	—
Item D3.1	—	0.913	0.872
Item D3.2	—	0.835	0.902
Item D3.3	—	0.796	0.921
Item D3.4	—	0.807	0.909
Getting along (D4)	0.965	—	—
Item D4.1	—	0.933	0.953
Item D4.2	—	0.921	0.954
Item D4.3	—	0.939	0.951
Item D4.4	—	0.889	0.962
Item D4.5	—	0.879	0.964
Housework (D5.1‐4)	0.978	—	—
Item D5.1	—	0.954	0.968
Item D5.2	—	0.936	0.973
Item D5.3	—	0.934	0.974
Item D5.4	—	0.954	0.968
Work/school activities (D5.5‐8)^a^	0.917	—	—
Item D5.5	—	0.817	0.889
Item D5.6	—	0.675	0.915
Item D5.7	—	0.862	0.875
Item D5.8	—	0.898	0.860
Participation(D6)	0.976	—	—
Item D6.1	—	0.921	0.972
Item D6.2	—	0.900	0.973
Item D6.3	—	0.868	0.975
Item D6.4	—	0.897	0.973
Item D6.5	—	0.926	0.972
Item D6.6	—	0.934	0.971
Item D6.7	—	0.925	0.972
Item D6.8	—	0.870	0.975
Total score (32‐Items)	0.990	—	—
Total score (36‐Items)	0.981	—	—

Abbreviations: *α*, Cronbach's alpha; D, domain; WHODAS‐2, World Health Organization Disability Assessment Schedule‐II.

^a^ Domain 5.5‐8 work/school activity was tested in 26 participants.

The 12‐item WHODAS 2.0 and the 11‐item WHODAS (ignoring work/school activity) had Cronbach's alpha coefficients of 0.952 and 0.973, respectively. Internal consistency of the 12 items within the domain was evaluated using the Spearman‐Brown coefficient (split half), which was 0.916 for cognitive, 0.929 for mobility, 0.926 for self‐care, 0.874 for life activities, 0.914 for participation, and 0.940 for getting along with others.

### 3.5 Floor and Ceiling Effect

Floor effects were found in all domains: 57%, 40%, 55%, 56%, 57%, 47%, and 45% in Domain 1, Domain 2, Domain 3, Domain 4, Domain 5a, Domain 5b, and Domain 6, respectively, whereas ceiling effects were not present in any domain and ranged from 0.0% to 0.88%.

### 3.6 Construct Validity

We present the correlations between the short‐form (12‐item) and full‐form (36‐item) WHODAS 2.0 assessments across various domains of functioning. The correlations demonstrated the degree of agreement between the two versions in measuring different aspects of functioning and disability, including cognition, self‐care, getting around, getting along with others, life activities, and participation. Overall, the results indicated strong correlations across most domains, suggesting that the short‐form WHODAS 2.0 is a valid alternative to the full version for assessing disability. High correlations were observed in the domains of cognition (D1), mobility (D2), self‐care (D3), getting along (D4), life activity (D5a), and participation (D6) with coefficients exceeding 0.909, indicating that both versions effectively capture this aspect of functioning. The total WHODAS 2.0 scores revealed a high correlation of 0.990, further supporting the validity of the short‐form as a comprehensive tool for evaluating overall functioning (Table 4).

**Table 4:** Correlations of the full‐form (36‐item) and short‐form (12‐item) WHODAS 2.0.

	36‐item WHODAS 2.0 (full version)							
12‐items WHODAS 2.0 (short form)	Understanding and communicating (D1.1–1.6)	Getting around (D2.1–2.5)	Self‐care (D3.1–3.4)	Getting along (D4.1–4.5)	Life activities in		Participation (D6.1–6.8)	Total WHODAS 2.0 score
					House (D5.1–5.4)	Work/school (D5.5–5.8)		
S3 and S6 (cognition)	**0.970**	0.826	0.771	0.822	0.812	0.346	0.852	0.916
S1 and S7 (Mobility)	0.727	**0.972**	0.810	0.767	0.836	0.406	0.827	0.897
S8 and S9 (Self‐care)	0.739	0.840	**0.970**	0.780	0.817	0.445	0.804	0.873
S10 and S11 (Getting along)	0.827	0.785	0.748	**0.981**	0.798	0.445	0.804	0.879
S2 (Life‐activities)	0.768	0.829	0.785	0.858	**0.966**	0.539	0.839	0.890
S12 (Life‐activities)	0.598	0.680	0.539	0.793	0.796	**0.909**	0.909	0.776
S4 and S5 (Participation)	0.797	0.847	0.820	0.835	0.858	0.434	**0.971**	0.929
Overall D12	0.883	0.936	0.887	0.912	0.911	0.723	0.925	**0.990**

*Note:* Values in bold represent the correlations between each short‐form domain and its corresponding domain in the full 36‐item WHODAS 2.0.

Abbreviation: WHODAS‐2, World Health Organization Disability Assessment Schedule‐II.

A correlation matrix between the six domains and the overall score of the 36‐item WHODAS 2.0 was developed to assess the validity of domain selection. The correlation level was strong in each case and statistically significant at *p* < 0.001. Very strong correlations were observed between the domains and the overall domain score of functioning and disability (*r* = 0.871–0.944). Strong correlations were also observed between the domains of functioning and disability (*r* = 0.729–0.862) (Table 5).

**Table 5:** Correlations of the WHODAS 2.0 with all the domains and their summary scores.

Domains	D1	D2	D3	D4	D5	D6	Overall score
D1: Cognition	1	—	—	—	—	—	—
D2: Mobility	0.807	1	—	—	—	—	—
D3: Self‐care	0.729	0.835	1	—	—	—	—
D4: Getting along	0.840	0.813	0.761	1	—	—	—
D5: Life activities	0.793	0.855	0.810	0.836	1	—	—
D6: Participation	0.837	0.857	0.822	0.837	0.862	1	—
Overall score	**0.907**	**0.936**	**0.871**	**0.905**	**0.915**	**0.944**	**1**

*Note:* Values in bold represent the correlations between each domain and overall score.

Abbreviations: D, domain; WHODAS‐2, World Health Organization Disability Assessment Schedule‐II.

We investigated the WHODAS 2.0's theoretical validity by analyzing its internal structure, the correlations between the items in the domain and the domain itself, and the correlations between the domain's items and the overall disability score. Results showed that the domain result and the overall result had strong correlation (*r* = 0.817–0.966 and *r* = 0.629–0.927, respectively). All statistical analysis results were significant at *p* < 0.01 (Table 6).

**Table 6:** Correlations between WHODAS 2.0 items, domains, and the overall results.

Domain	Domain items	Domain results	Overall results
Domain 1: Cognition	Item 1	0.927	0.919
	Item 2	0.953	0.892
	Item 3	0.939	0.858
	Item 4	0.935	0.848
	Item 5	0.916	0.797
	Item 6	0.885	0.783
Domain 2: Mobility	Item 1	0.949	0.871
	Item 2	0.930	0.885
	Item 3	0.935	0.898
	Item 4	0.939	0.861
	Item 5	0.931	0.867
Domain 3: Self‐care	Item 1	0.956	0.864
	Item 2	0.930	0.829
	Item 3	0.875	0.759
	Item 4	0.911	0.798
Domain 4: Getting along	Item 1	0.945	0.863
	Item 2	0.943	0.839
	Item 3	0.944	0.859
	Item 4	0.939	0.842
	Item 5	0.919	0.877
Domain 5a: Life activity	Item 1	0.966	0.890
	Item 2	0.963	0.893
	Item 3	0.963	0.881
	Item 4	0.963	0.882
Domain 5b: Life activity^a^	Item 5	0.909	0.776
	Item 6	0.817	0.629
	Item 7	0.892	0.778
	Item 8	0.929	0.717
Domain 6: Participation	Item 1	0.902	0.881
	Item 2	0.894	0.816
	Item 3	0.865	0.838
	Item 4	0.904	0.844
	Item 5	0.934	0.887
	Item 6	0.937	0.927
	Item 7	0.944	0.925
	Item 8	0.906	0.849

Abbreviation: WHODAS‐2, World Health Organization Disability Assessment Schedule‐II.

^a^ Domain 5b: Life activity number of participants = 26.

### 3.7 CFA

**Figure 1:**
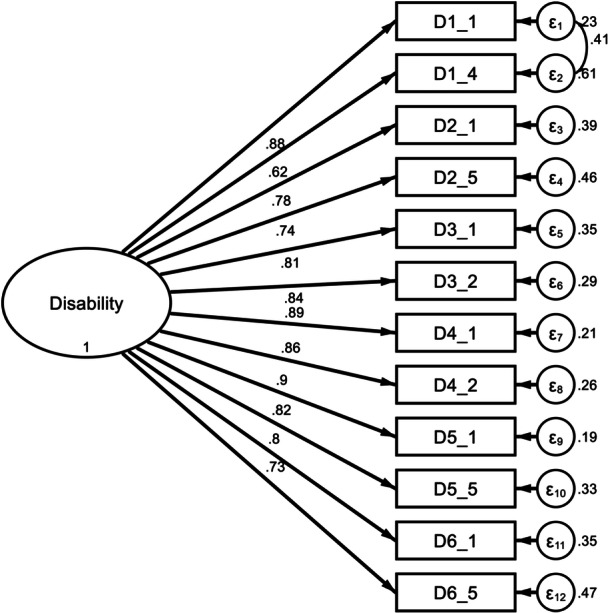
A one‐factor structure diagram of the confirmatory factor analysis for WHODAS 2.0 in participants with PLTC. PLTC, potentially life‐threatening conditions; WHODAS‐2, World Health Organization Disability Assessment Schedule‐II.

**Figure 2:**
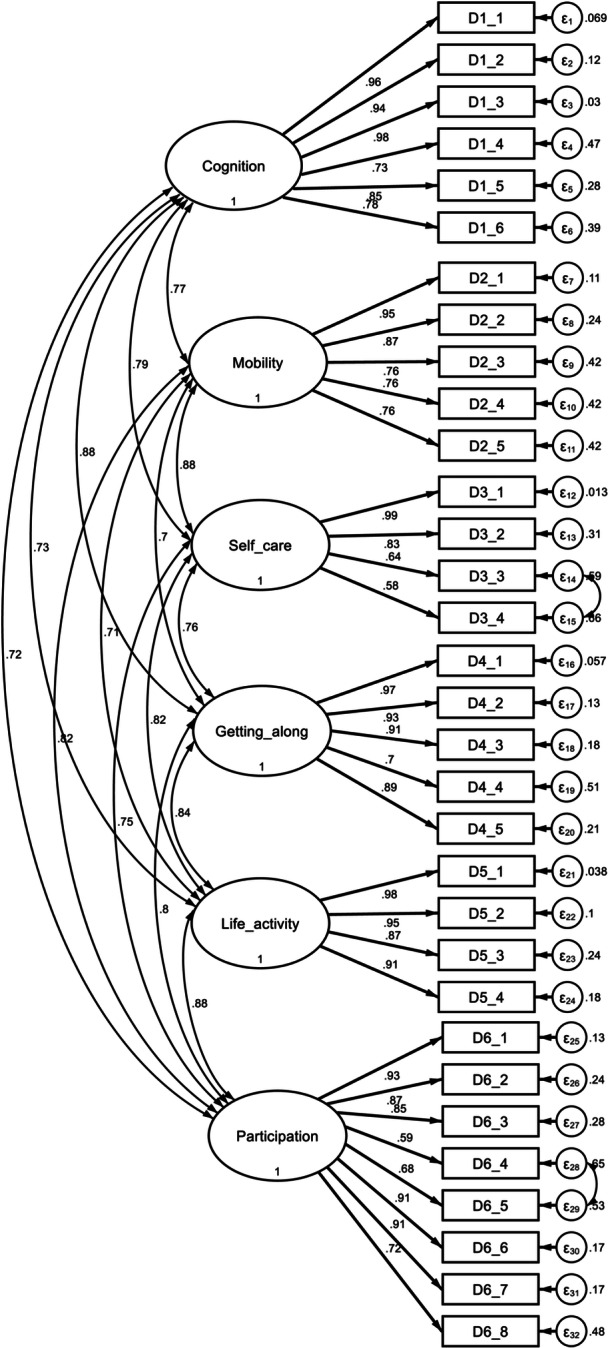
Six‐factor structure diagram of the confirmatory factor analysis for WHODAS 2.0 in participants with PLTC. PLTC, potentially life‐threatening conditions; WHODAS‐2, World Health Organization Disability Assessment Schedule‐II.

The KMO test, which measures sample adequacy, and Bartlett's test of sphericity were used to check whether the data was suitable for factor analysis. KMO = 0.939, Bartlett's test of sphericity < 0.001, and a total variance of 81.70% were the results shown. CFA was conducted using both a one‐factor structure (short form) and a six‐factor structure (full version). For both models, the goodness‐of‐fit indices fell outside the acceptable range. For instance, the one‐factor model exhibited the following fit indices: RMSEA = 0.300, SRM*R* = 0.084, CFI = 0.682 and TLI = 0.604. Factor loadings in the one‐factor structure ranged from 0.62 (D2.1) to 0.90 (D5.1) (Figure 1), while those in the six‐factor structure ranged from 0.58 (D3.4) to 0.99 (D3.1) (Figure 2).

## 4 Discussion

In both public health research and clinical practice, health scales have gained significant popularity [28]. According to the WHO, evaluating health requires consideration of functional, psychological, and social dimensions, reflecting its complex and multifaceted nature. Health is difficult to evaluate since it is multidimensional and dependent on both internal and environmental influences. To evaluate health with a comprehensive and holistic approach, it is necessary to develop scales that assess different aspects of health conditions. Furthermore, for any research tool to be effective, it must demonstrate high validity and reliability in its measurements. If scales from one culture are used in another, adaptations are needed because a reliable measure of suffering in one culture may not be significant or understandable in another [29].

With certain limitations, the WHODAS 2.0 Tigrigna version confirmed acceptable scale properties. The Cronbach's alpha values of the total score and all the domains had excellent internal consistency (alpha ≥ 0.90), except for the self‐care (D3) and work/school activities (D5b), which still maintained good internal consistency with an alpha value of > 0.860. It also satisfies Nunnally's requirement, according to which Cronbach's alpha for a good scale must be more than 0.70 [19]. We verified that the 36‐item WHODAS 2.0 had good reliability in our study of women with PLTC. Compared with the other domains, the “self‐care” and work/school activity domains have slightly lower reliability values, which is not unique to our study [30, 31]. The internal consistency reliability of the WHODAS 2.0 from the general population and specific patient populations worldwide showed similar results, as the lower alpha values in the cited article were found for self‐care and work/school activity [32, 33, 34]. The reasons for lower internal consistency in the “self‐care” and work/school activities domain could be due to the small number of items in the domain. WHODAS 2.0 has fewer items in self‐care (4 items) and work/school (4 items) compared to other domains (e.g., mobility has 5, cognition has 6, participation has 8). Cronbach's alpha is sensitive to scale length, and shorter scales often yield lower reliability estimates [35].

The strong correlation (*r* = 0.990) between the 36‐item WHODAS 2.0 and the total score of the 12‐item suggests excellent convergent validity. Thus, indicating that the short form version effectively captures the overall disability construct measured by the full version. In the specific domains, it also indicates that there were strong correlations (cognition: *r* = 0.970, mobility: *r* = 0.972, self‐care: *r* = 0.970, getting along with: *r* = 0.981, life activities: *r* = 0.966, and participation: *r* = 0.971) between short version items and their corresponding full form domains, indicating strong convergent validity. This study aligns with other studies conducted by WHO that show the 12 items capture around 80% of the variance in the full version [12].

The results support the divergent validity of the short‐form WHODAS 2.0, with low correlations between theoretically distinct domains compared to strong within‐domain associations. This aligns with the instrument's design, which aims to differentiate between distinct functional areas while maintaining interconnected overall disability measure [12, 36]. These findings support the utility of the short‐form WHODAS 2.0 for both domain‐specific and overall disability assessments in clinical and research settings [36]. In confirmatory factor analysis, strong factor loadings show that the observed variables are adequately represented by latent factors. However, poor model fit (such as high RMSEA and low CFI/TLI) suggests that the overall model does not adequately represent the data. The small sample size may be the cause of this poor model fit. A small sample size can lead to unstable estimates and inflated factor loadings while also negatively impacting fit indices [37].

Although the KMO index (KMO = 0.939) and Bartlett's test of sphericity (< 0.001) indicated sufficient sample adequacy for factorability, these measures do not fully compensate for the limitations imposed by a small sample size, which may affect the stability of the factor structure. With this limitation, our results indicate positive findings about the validity and reliability of the Tigrigna version of the WHODAS 2.0. Therefore, we suggest that the Tigrigna version of the WHODAS 2.0 is valid for measuring functioning and disability of PLTCs in Tigray, northern Ethiopia. Validating the WHODAS 2.0 tool for PLTCs has been performed, which improves its use in analyzing long‐term functional disability, guiding future research on postpartum recovery, improving maternal health frameworks, guiding public health policies, and informing targeted clinical interventions.

Concerning the study's limitations, CFA analysis is more sample dependent to confirm the validity of unresolved subscales and enable the development of a valid scoring structure, as the one we applied in this study could be sample dependent, especially owing to the floor effect in every item and a consequence of the low response rate in some categories. Another limitation is the absence of a test–retest assessment; the current study was cross‐sectional.

## 5 Conclusion

This study evaluated the validity and reliability of the Tigrigna version of WHODAS 2.0 in assessing disability among women who experienced PLTC in Tigray, Ethiopia. The findings indicate that the tool has excellent internal consistency for most domains (Cronbach's *α* > 0.90), strong convergent validity (high correlation between 36‐item and 12‐item versions, *r* = 0.990), and acceptable content validity (I‐CVI = 0.78–1.0, S‐CVI = 0.96). However, confirmatory factor analysis (CFA) revealed poor model fit, likely due to the small sample size, which may have led to unstable estimates and inflated factor loadings. Despite this limitation, the Tigrigna WHODAS 2.0 effectively captures functional disability in women with PLTCs. Its adoption in clinical practice and research can enhance postpartum care and disability monitoring. Further studies with larger samples and longitudinal designs are needed to optimize its use.

## Author Contributions

**Fitiwi Tinsae Baykemagn:** conceptualization, investigation, writing–original draft, methodology, validation, visualization, formal analysis, project administration, data curation, supervision, resources, funding acquisition, software, writing – review and editing. **Girmatsion Fisseha Abreha:** conceptualization, methodology, writing – review and editing. **Yibrah Berhe Zelelow:** conceptualization, writing–review and editing, validation, methodology. **Alemayehu Bayray Kahsay:** conceptualization, methodology, writing – review and editing.

## Funding

The authors have nothing to report.

## Ethics Statement

Ethical approval was obtained from the Institutional Review Board (IRB) of Mekelle University, college of health sciences (approval reference: MU‐IRB 2129/2023), which holds authority for ethical approval of health research in the Tigray region. The IRB approval explicitly covered pre‐consent access to patient contact information for this study, as well as the subsequent medical record review, and was obtained prior to commencing the review. The study accessed hospital registration books and medical charts at Lemlem Karl hospital, Ayder Comprehensive Specialized Hospital, Mekelle hospital, Wukro hospital, Adigrat hospital, Abi‐adi hospital, Adwa hospital, St Mary hospital, Aksum Comprehensive Specialized Hospital, and Suhul hospital in the Tigray region. These hospitals are independent entities directly affiliated with and under the oversight of the Tigray Regional Health Bureau. To ensure proper authorization for data access, a written supporting letter was obtained from the TRHB permitting the study and data access across affiliated hospitals, and institutional permission was also obtained from the administration of each participating hospital prior to data collection.

## Consent

Verbal informed consent was obtained from each participant. The consent information was read aloud, questions were addressed, and verbal agreement was documented on the consent form before proceeding. Written informed consent was not required, per approval from the IRB, in light of literacy challenges in Ethiopia, including lower female literacy rates (around 48% for women aged 15–49). This method was deemed appropriate to respect participant autonomy without imposing barriers related to reading or signing.

## Conflicts of Interest

The authors declare no conflicts of interest.

## Data Availability

The data that support the findings of this study are available from the corresponding author upon reasonable request. When a reasonable request is made, the corresponding author can provide the dataset used and/or analyzed in this study.
